# Do Arctic breeding geese track or overtake a green wave during spring migration?

**DOI:** 10.1038/srep08749

**Published:** 2015-03-04

**Authors:** Yali Si, Qinchuan Xin, Willem F. de Boer, Peng Gong, Ronald C. Ydenberg, Herbert H. T. Prins

**Affiliations:** 1 Ministry of Education Key Laboratory for Earth System Modeling, and Center for Earth System Science, Tsinghua University, Qinghuayuan 1, 100084, Beijing, China; 2 Department of Animal and Plant Sciences, Alfred Denny Building, University of Sheffield, S10 2TN Sheffield, UK; 3 Resource Ecology Group, Wageningen University, Droevendaalsesteeg 3a, 6708 PB Wageningen, The Netherlands; 4 Department of Biological Sciences, Centre for Wildlife Ecology, Simon Fraser University, V5A 1S6 Burnaby, BC, Canada

## Abstract

Geese breeding in the Arctic have to do so in a short time-window while having sufficient body reserves. Hence, arrival time and body condition upon arrival largely influence breeding success. The green wave hypothesis posits that geese track a successively delayed spring flush of plant development on the way to their breeding sites. The green wave has been interpreted as representing either the onset of spring or the peak in nutrient biomass. However, geese tend to adopt a partial capital breeding strategy and might overtake the green wave to accomplish a timely arrival on the breeding site. To test the green wave hypothesis, we link the satellite-derived onset of spring and peak in nutrient biomass with the stopover schedule of individual Barnacle Geese. We find that geese track neither the onset of spring nor the peak in nutrient biomass. Rather, they arrive at the southernmost stopover site around the peak in nutrient biomass, and gradually overtake the green wave to match their arrival at the breeding site with the local onset of spring, thereby ensuring gosling benefit from the peak in nutrient biomass. Our approach for estimating plant development stages is critical in testing the migration strategies of migratory herbivores.

Spring migration is one of the most essential parts of the annual life cycle of migratory birds. The decisions taken at stopover sites determine the refuelling potential and the arrival time at the breeding site, and, accordingly, greatly influence breeding success^1^,^2^,^3^. Bird migration is principally driven by an internal clock under photoperiodic control^4^ and fine-tuned in response to environmental factors, such as food supply, temperature, precipitation and wind conditions^5^,^6^,^7^,^8^,^9^. Since the photoperiod is fundamentally consistent year to year, whereas environmental conditions vary, it is advantageous for birds to be able to modify their activities to react to current circumstances^3^.

Arctic breeding geese have a narrow time window to breed. Arriving too late at the breeding site leads to a severe reduction in reproductive success or even complete breeding failure, due to the competition for high-quality territories and the time constraints to raise young to fledging^3^,^10^,^11^,^12^,^13^. However, arriving too early, when environmental conditions are still adverse and geese risk food shortage or cold snaps, is also costly^3^,^14^,^15^. Arctic breeding geese are therefore under natural selection pressure to arrive in the Arctic as soon as environmental conditions allow (i.e., when snow starts to melt at the onset of spring), and are selected against arriving too early or too late.

The advantage of early breeding is substantial, but egg laying is mainly constrained by body stores^1^,^2^,^3^. Accumulating nutrient stores along the flyway to compensate the scarcity upon arrival at the breeding site is an advantageous strategy. Females that bring the nutrients used for egg production along to the breeding grounds are considered ‘capital breeders’ whereas those that obtain these nutrients locally after arrival are referred to as ‘income breeders’. Geese combine these strategies and accumulate body stores along the flyway to be able to start breeding shortly after securing a territory^16^,^17^,^18^,^19^. This strategy enables Arctic-breeding geese to reserve the peak of local food resources for their young^1^,^20^.

The green wave hypothesis posits that migratory geese track a successively delayed spring flush of plants on their way from the wintering grounds in the temperate zone to their Arctic breeding site^6^,^21^. Previous studies investigating this food effect mostly interpreted the green wave as the onset of spring^22^,^23^,^24^,^25^,^26^,^27^, whereas a field study^28^ demonstrated that the peak in nutrient biomass (i.e., plants with the highest amount of nitrogen per unit area) is a key factor driving the timing of spring migration, but that geese skip ahead of this peak at the breeding site to benefit gosling rearing later in the season.

Despite the pronounced effect of the onset of spring in timing the spring migration of geese suggested by previous studies^23^,^25^, a non-significant effect of the onset of spring in timing the arrival of geese was reported by Eichhorn et al. (2009)^27^ and geese managed to breed within the short time window at their breeding site regardless of an early or late departure from their wintering sites^27^. Moreover, geese were found to stage during early spring, when breeding site was still snow covered, at suitable sites close by to gain body stores by grazing on emerging plants, and to facilitate a timely arrival at the breeding site^29^. Hence, we suggest that geese might not track any specific development stage of spring growing plants (either the onset of spring or the peak in nutrient biomass) along the flyway but rather overtake the green wave. The validity of this overtaking of the green wave has yet to be investigated in a natural setting along a flyway, using detailed plant phenology data and individual goose migration data.

An accurate estimation of the plant green-up along the flyway is critical in testing the green wave hypothesis. Studies that interpreted the green wave as the onset of spring, often quantified the green wave using a proxy of plant phenology, namely “growing degree days” (GGD) or its derivatives calculated from the accumulated temperature above a pre-defined threshold^22^,^23^,^27^,^30^. Vegetation indices derived from satellite imagery have been used to offer a more direct and detailed measurement of plant phenology. However, only relatively coarse-resolution vegetation indices and arbitrary thresholds/ranges have been used^25^,^26^,^31^.

Measuring the peak in nutrient biomass in the field is time-consuming^28^ and this specific stage of plant development has yet to be estimated along the migratory route. Given that plant biomass increases and digestibility decreases after the onset of spring growth, the peak in nutrient biomass corresponds to immature plants with an intermediate development stage, which offer the optimal intake rate of digestible nutrient as predicted by the forage maturation hypothesis^32^,^33^,^34^,^35^,^36^,^37^. Satellite imagery with a finer spatial and temporal resolution in combination with a change rate method could be used to estimate these specific plant development stages and further investigate their influence on the spring migration schedule of Arctic breeding geese.

In this study we test the role of plant phenology in the timing of arrival of individual Barnacle Geese *Branta leucopsis* at the stopover and the breeding sites during their spring migration on the way from the Wadden Sea in Western Europe to their breeding site in Arctic Russia. We firstly calculate the timing of the green wave (defined as either the onset of spring or the peak in nutrient biomass), based on the change rate of the herbaceous-plant phenology derived from satellite imagery. Next we extract the stopover schedules of individual Barnacle Geese from a published dataset^38^. We then test whether individual Barnacle Geese follow a specific plant development stage (i.e., the onset of spring or the peak in nutrient biomass) during spring migration or overtake the green wave and arrive at the breeding site at the local onset of spring. We use the root mean square deviation (RMSD) method to examine the difference between goose arrival time and the time of the onset of spring and the peak in nutrient biomass and then compare the plant development level at goose arrival time across different sites using a generalized linear-mixed model (GLMM).

## Results

**Figure 1:**
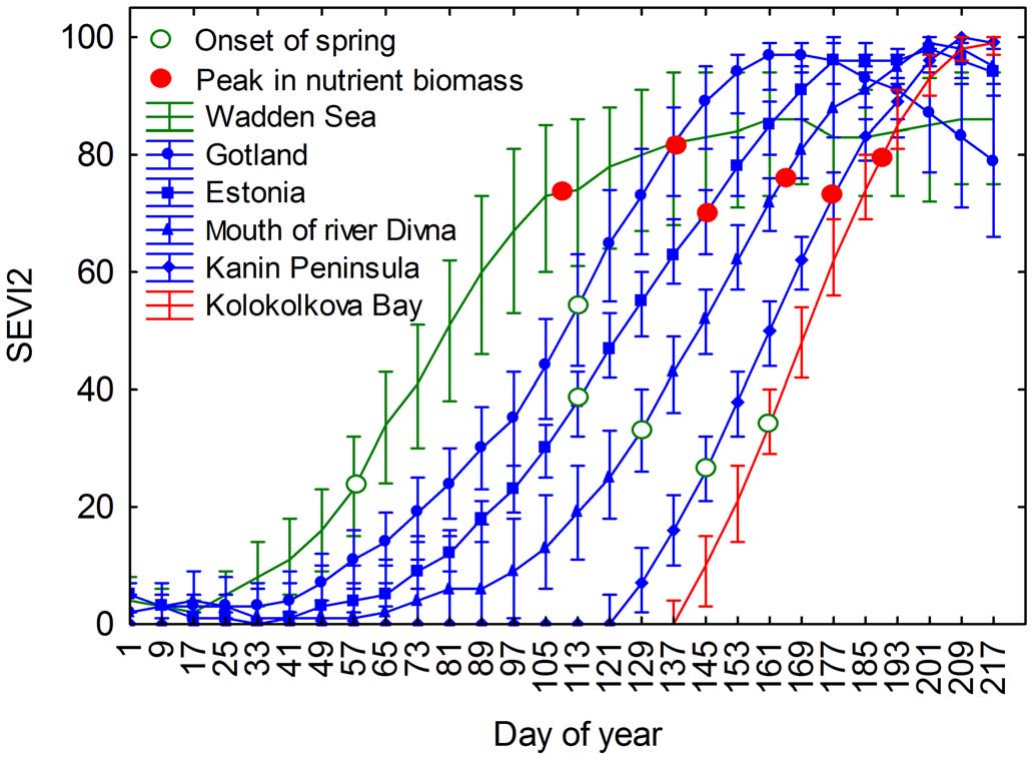
Progression of herbaceous plant development at the wintering (green), the stopover (blue) and the breeding site (red) of Barnacle Geese. Mid-points indicate the median of the standardized two-band Enhanced Vegetation Index (SEVI2) at each site and specific time point, and upper and lower bars indicate the 25 and 75 percentiles of the non-outlier range. Open green and filled red dots indicate the date of the onset of spring and the peak in nutrient biomass.

The phenology development of herbaceous plants in the wintering, stopover, and breeding sites of Barnacle Geese was quantified by a standardized two-band Enhanced Vegetation Index (SEVI2) (Fig. 1). A successively delayed onset of spring (day of year: 57, 113, 113, 129, 145, and 161) and peak in nutrient biomass (day of year: 109, 137, 145, 165, 177, and 189) was observed from the wintering site of Barnacle Geese in the Wadden Sea located in the temperate zone, via the four main stopover sites in Gotland, Estonia, the mouth of the river Divna, and the Kanin Peninsula, to the Arctic breeding site Kolokolkova Bay.

**Figure 2:**
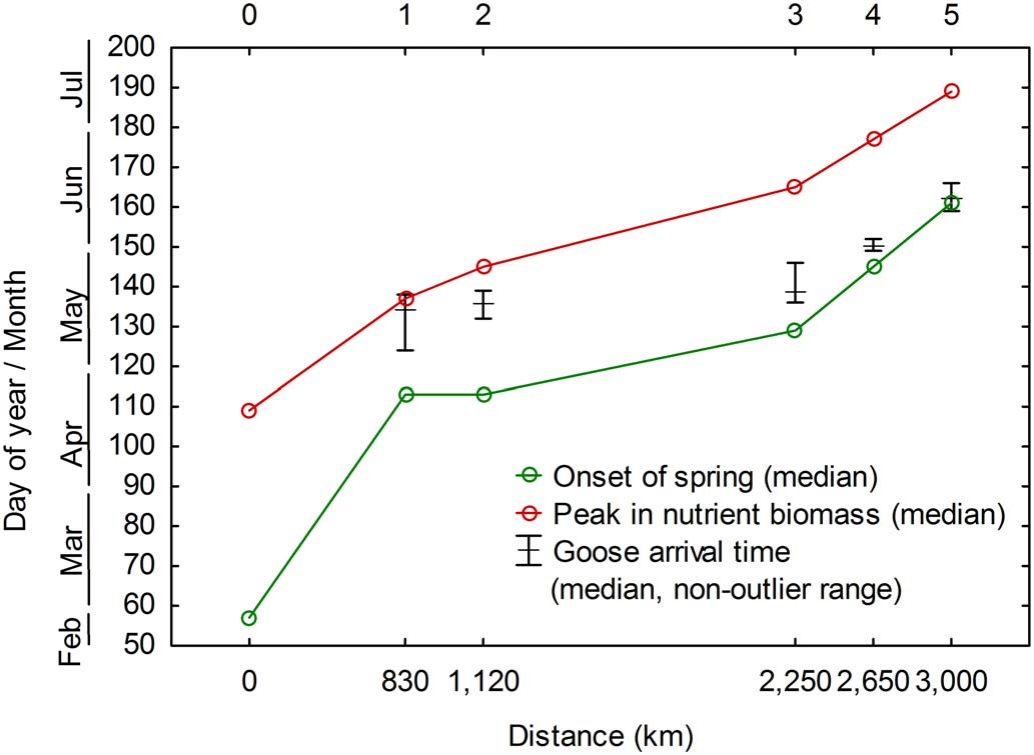
Dates of the onset of spring and the peak in nutrient biomass at each site against the flight distances. Sites include wintering site (0), stopover sites (1–4), and the breeding site (5). Error bars indicate the median and range of the arrival time of 19 female Barnacle Geese derived from Eichhorn et al. (2006)^38^.

**Figure 3:**
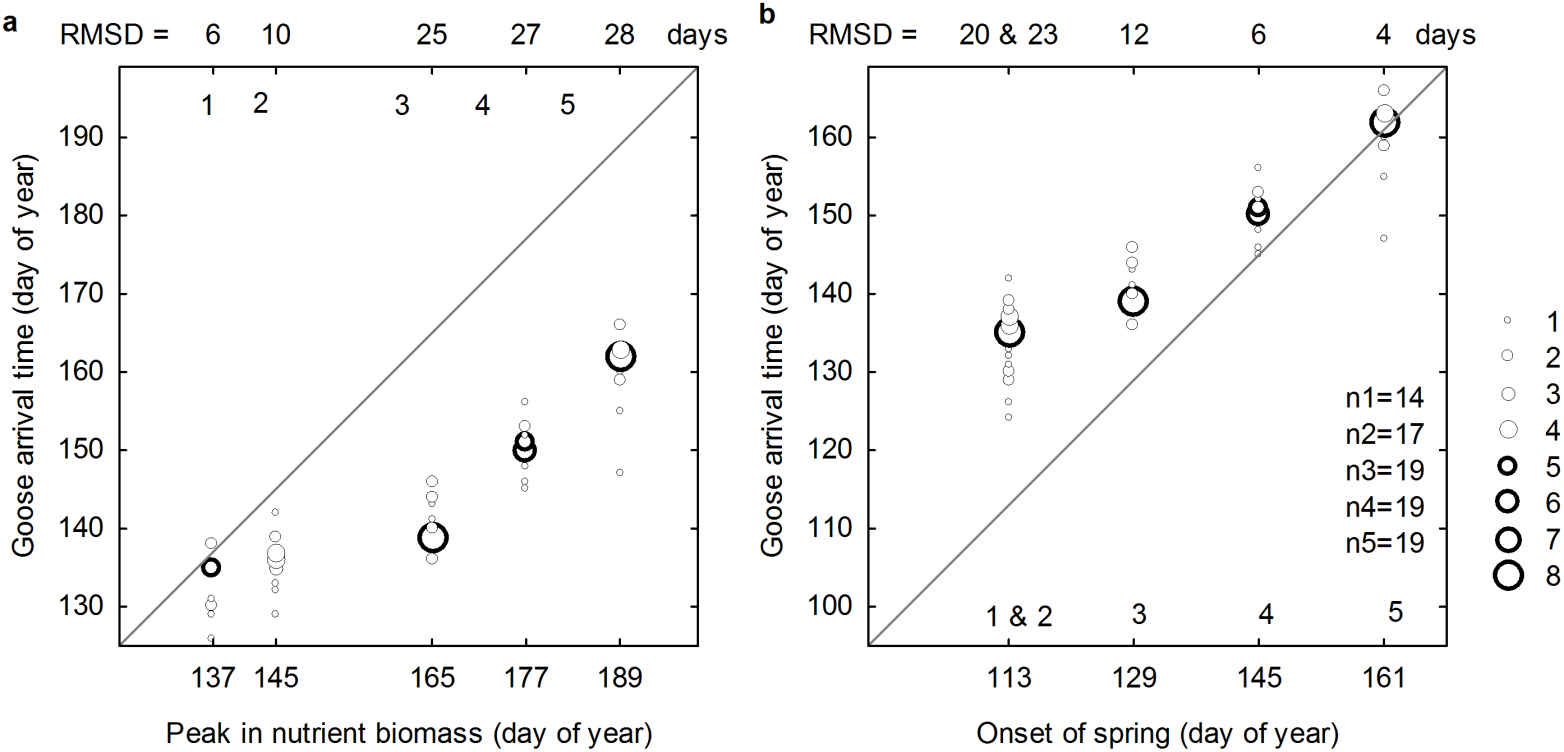
The match between goose arrival time during spring migration and the date that plants reach a specific development stage. Specific plant development stages are the peak in nutrient biomass (a) and the onset of spring (b). RMSD and the number of observations (n) are indicated for each stopover site (1–4) and the breeding site (5).

The timing of goose arrival along the flyway gradually shifted from close to the peak in nutrient biomass (RMSD = 6 days) to a good match with the onset of spring (RMSD = 4 days) at the breeding site (Fig. 2 and 3). The timing of geese arriving at the southern stopover sites (1 – Gotland and 2 – Estonia) was later than the onset of spring and approached the peak in nutrition biomass, whereas the timing of geese arriving at the northern sites (3 – the mouth of the river Divna, 4 – the Kanin Peninsula, and 5 – the Kolokolkova Bay) was closer to the onset of spring and before the peak in nutrition biomass. Accordingly, geese arrived at different sites at different levels of plant development during their migration.

As migration progressed from the southern stopover site 1 to the breeding site 5, we found an increasing difference with the peak in nutrient biomass (RMSD = 6, 10, 25, 27 and 28) (Fig. 3a) and a decreasing difference between the goose arrival time and the onset of spring (RMSD = 20, 23, 12, 6, and 4 days) (Fig. 3b). The goose arrival time at the breeding site Kolokolkova Bay (RMSE = 4 days) yielded the highest match with the local onset of spring in comparison to the fit with any stage of plant development at any other site during spring migration.

**Figure 4:**
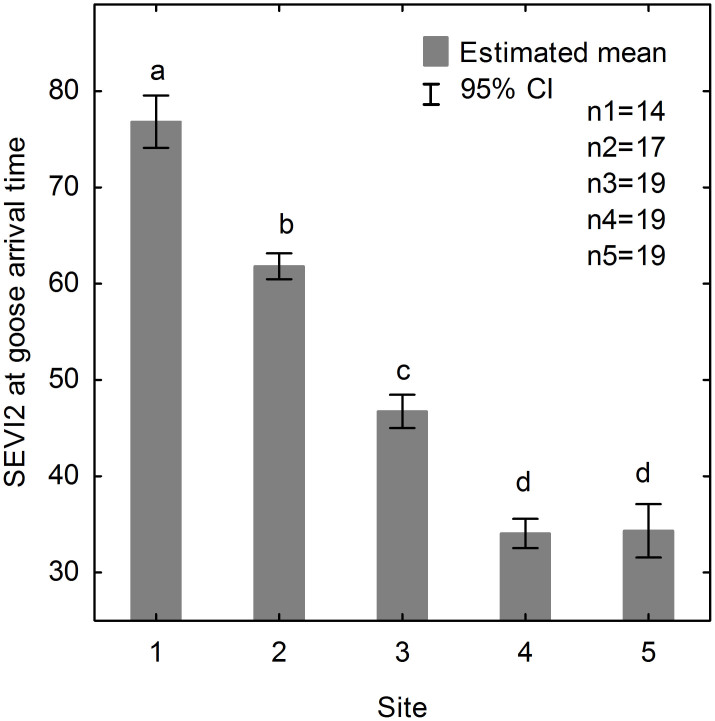
Standardized two-band Enhanced Vegetation Index (SEVI2) at goose arrival time across the stopover sites (1–4) and the breeding site (5). Solid grey bars represent the estimated mean and error bars represent 95% confident interval (CI) predicted by the generalized linear mixed model (GLMM) (*F* = 467.187, *df1* = 4, *df2* = 83, *P* < 0.001). Different letters indicate significant differences between paired sites. Pairwise comparisons from sequential Bonferroni tests show *P* = 0.827 for site 3 and 4, and *P* < 0.001 for other paired sites (see Supplementary Table S2 online).

The fixed-effect factor ‘site’ had a significant effect on the plant development level at goose arrival time as predicted by a GLMM (*F* = 467.187, *df1* = 4, *df2* = 83, *P* < 0.001). The plant development level at goose arrival time showed a significant lower level along the flyway from the southernmost stopover site 1 to the northernmost stopover site 4 (sequential Bonferroni tests: *P* < 0.001) (Fig. 4). Similar plant development levels were found in the stopover site 4 and the breeding site 5 (sequential Bonferroni tests: *P* = 0.827) (Fig. 4). Geese followed the successively lower plant development levels before they reached the breeding site 5.

## Discussion

This study utilizes specific plant phenology development patterns derived from relatively high spatiotemporal resolution satellite imagery, combined with a reconstruction of migration timing deduced from Barnacle Geese tracking data, to test whether geese track or overtake a green wave during spring migration. We demonstrate that Barnacle Geese track neither the onset of spring nor the peak in nutrient biomass on their way to the breeding site. Rather, they gradually overtake the green wave by arriving at the southernmost stopover site at a high plant development level (close to the peak in nutrient biomass), following the successively lower level of plant development as migration progresses, and matching their arrival at the breeding site with the local onset of spring, which facilitates gosling rearing later in the season. Although geese do forage along the flyway, the development stage of plants is secondary to their aim of timely arrival at the breeding site.

Barnacle Geese do not follow the onset of spring on the way to their Arctic breeding site. We find a decreasing difference between the goose arrival time and the onset of spring as geese move northwards, running from stopover sites with a poor match (e.g., at Gotland RMSD = 20 days and at Estonia RMSD = 23 days) to a reasonable match at the point that geese reached the breeding site (RMSD = 4 days). A previous study^23^ compared different decision rules (e.g., body stores, photoperiod, and temperature) and reported a pronounced effect of the onset of spring in timing the departure of Pink-footed Geese *Anser brachyrhynchus* during spring migration. However, the match between their observed and simulated departure dates based on the onset of spring rule show a poorer fit at the southern stopover site (Denmark RMSD = 14 days) than the northern ones (mid Norway RMSD = 8 days and North Norway RMSD = 6 days), which is consistent with our findings, and suggests that the onset of spring was not consistently tracked by these geese. Furthermore, the difference between the migration timing of Barnacle Geese and the onset of spring at the wintering site in the Wadden Sea has increased since the 1990s (geese delayed their departure from their wintering site) due to the competition for food and predation risk in the Baltic region^27^,^39^.

Our estimation of the peak in nutrient biomass derived from satellite imagery (day 109 at the Wadden Sea and day 137 at Gotland) is consistent with the ground reference data measured during the same spring (day 112 at the Wadden Sea and day 145 at Gotland)^28^. Based on the goose movement from the Wadden Sea to Gotland, a previous study^28^ suggested that geese leave their current site as the food passes the peak in nutrient biomass, and arrive at the next site the moment food reaches this optimal condition. However, our results for the subsequent stopover sites do not support this statement. Although a fair match between the arrival time of geese and the peak in nutrient biomass is observed at Gotland (RMSD = 6 days), the difference increases as spring migration progresses (e.g. at Estonia RMSD = 10 days and at the mouth of the river Divna RMSD = 25 days) (Fig. 2 and 3). Accordingly, a strategy of tracking the peak in nutrient biomass is not followed by these Barnacle Geese.

In agreement with van der Graaf et al. (2006)^28^, our results support that geese arrive early at the breeding site prior to the flush of spring growth of plants. We specifically define the early arrival as the time of the local onset of spring. Our findings underline the partial capital breeding strategy adopted by Barnacle Geese^1^,^19^,^20^: these geese take advantage of the peak in nutrient biomass at the southernmost stopover site but, while overtaking the green wave, arrive at the breeding site at the local onset of spring, well before the time of the peak in nutrient biomass. Based on our estimation of plant phenology, the time of the peak in nutrient biomass at the breeding site is 28 days after geese arrival (Fig. 3). Compared with the mean laying date of geese (3 days after their arrival)^38^ and the incubation period (25 days)^40^, the gosling rearing period coincides with the time of the peak in nutrient biomass. Hence, the arrival strategy of the Barnacle Geese allows their goslings to benefit from the peak in nutrient biomass later in the season and optimizes goslings’ chance of fledging.

We found a relatively good match between goose arrival time and the onset of spring at the most northern stopover site Kanin Peninsula (RMSD = 6 days). The plant development level at goose arrival time also indicated a significant lower plant development level at the site Kanin Peninsula in comparison to the other stopover sites (*P* < 0.001), but a similar level with the breeding site (*P* = 0.827). This finding is in agreement with Hubner (2006)^29^, who observed that geese arrive at sites close to their breeding site (Varsolbukta for Spitsbergen Barnacle Geese) when snow starts to melt and plants start to grow. Geese staging at these sites manage to accumulate significant amount of body stores during their stay before moving to their breeding site^29^,^38^. This strategy of geese staying in close proximity of their breeding site facilitates a timely arrival at the breeding site, as soon as environmental conditions permit. Hence geese allow for some flexibility in response to local conditions, while increasing body stores in the meantime.

In this study we used relatively rough tracking data of 19 Barnacle Geese combined with observational data obtained during the spring migration season. Since this group of Barnacle Geese comprised a random draw of female breeders in the Tobseda colony, we posit that our results are in general true for the colony. This study used single-year goose migration and plant phenology data. We compared the plant phenology at the stopover and breeding sites in year 2004 with the long-term situation (2001–2010) and found that this year fell into the 25%–75% non-outlier range (see Supplementary Fig. S3 online), meaning 2004 was a representative year of plant phenology. Using multiple-year goose migration data (obtained via e.g., satellite tracking data) is recommended for further validation of the findings of this study.

A detailed characterization of the green wave is critical in assessing whether geese surf the wave (regardless of how it is defined) or whether they overtake it; whether they are driven by forage quantity, quality or profitability; and how well they can predict spatiotemporally dynamic conditions. This study proposes an approach to quantify specified levels of plant development along the migration flyway during the course of the spring growing season which can be used to test the spring migration strategy for other herbivores.

## Methods

### Prepossessing of satellite imagery

The Moderate Resolution Imaging Spectroradiometer (MODIS) Terra 8-day surface reflectance products MOD09Q1 (with a 250m spatial resolution) and yearly global land cover products (MCD12Q1) (with a 500m spatial resolution) were downloaded from http://e4ftl01.cr.usgs.gov/MODIS_Composites/MOLT/. The 8-day composite map was produced by assembling the most cloud-free pixels (the best pixels) within this 8-day time period into a single image. The spatial range of the imagery covers the whole migratory flyway and the time period covers the same year that the Barnacle Geese were tracked. A two-band Enhanced Vegetation Index (EVI2)^41^ was used to characterize plant phenology. EVI2 was calculated according to equation (1):
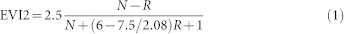
where N and R are surface reflectance in near-infrared and red bands.

There is an inherent level of noise unrelated to vegetation development in the MODIS time-series^42^,^43^,^44^. To reduce this noise, we eliminated the cloud contaminated observations based on the quality control data that comes with the satellite imagery and applied an adaptive Savitzky-Golay filter (with a window size of 6 and a polynomial degree of 2) to produce smoothed EVI2 time series^45^. Non-herbaceous land cover types were excluded based on the MODIS land cover map. We masked water, forest, urban and built-up area, snow and ice, barren and sparsely vegetated, and unclassified area from the SEVI2 maps. The remaining herbaceous plant area includes shrublands, savannas, grasslands, wetlands, croplands, and cropland and natural vegetation mosaic. All pixels with EVI2 values less than 0.05 (indicate non-vegetated areas like bare soil, water, snow, and ice) were set to 0.05^46^. To facilitate the comparison of plant development at different stopover sites, the EVI2 time series were standardized to a range of 0–100 by recalculating the original values according to equation (2):
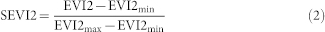
where EVI2_min_ and EVI2_max_ are the minimum and the maximum value of each EVI2 time series.

### Identifying the date of the onset of spring and the peak in nutrient biomass

For each stopover site (Gotland, Estonia, the mouth of the river Divna, and the Kanin Peninsula) and the breeding site at Kolokolkova Bay, a 50 km buffer zone was generated to represent the local plant development conditions. For each zone (site), the median of the SEVI2 values derived from herbaceous plant area was extracted from the 8-day interval maps, resulting in a SEVI2 time-series (with 46 median values) to describe the phenology development of each site. The median values were selected to minimize the effect of extremely low or high values related to cloud contamination or other unknown reasons. The change rate was calculated as (SEVI2_n+1_-SEVI2_n_)/8, where n ranges from 1 to 45 (change rate could not be calculated for the last value). A sudden increase in SEVI2 may signal the onset of substantial photosynthetic activity, which is defined as the beginning of plant growth^47^. Subsequently the onset of spring was estimated by identifying the maximum increase rate of the SEVI2 time series (medians) during the growing season. The peak in nutrient biomass (the intermediate plant development) was identified by calculating the mean date between the onset of spring and the date plant growth ceases (the change rate becomes ≤ 0 and SEVI2 starts to level off or decrease) (see Supplementary Fig. S1 online).

### Stopover patterns of Barnacle Geese

The stopover schedules of 19 female Barnacle Geese during 2004, tracked by Global Location Sensing (GLS) loggers and visual observations at the breeding site, were derived from Eichhorn et al. (2006)^38^. Geese left their wintering site in the Wadden Sea and stopped over along the flyway at Gotland, Estonia, the mouth of the river Divna, the Kanin Peninsula, before reaching their breeding site at Kolokolkova Bay (see Supplementary Fig. S2 online). The dates that individual geese (ID 1-19) arrived at each stopover site and the breeding site are shown in Supplementary Table S1 online. The area located north of the White Sea (i.e., two potential stopover sites – the mouth of the river Divna and the Kanin Peninsula, and the breeding site Kolokolkova Bay) fell outside the tracking system. For the breeding site at Kolokova Bay, 12 arrival dates were obtained by direct observations and 7 dates were filled by the mean date of the first sighting of another 80 ringed geese^38^. The arrival time of geese at the mouth of the river Divna was calculated by adding 1 day of flight after departing from the Baltic^38^. The arrival time at the Kanin Peninsula was derived as the midpoint between the calculated arrival at the mouth of river Divna and the (inferred and observed) arrival at the breeding site Kolokova Bay.

### Comparing goose arrival time with plant green-up level

The root mean square deviation (RMSD) method^48^ was used to examine the difference between goose arrival time and the specific plant development stages (i.e., the time of the onset of spring and the peak in nutrient biomass) at each stopover site and at the breeding site.

The RMSD was calculated according to equation (3):
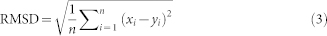
where n is the number of geese, x indicates the goose arrival time and y indicates the time when plants reach a specific development stage. To facilitate interpretation, a straight line described by y = x was drawn to show the fit between observed and predicted dates.

### Comparing plant development level at goose arrival time across sites

Linear interpolation was used to convert the 8-day interval plant phenology data into daily plant development curves. We then extracted the specific plant development level at each site at goose arrival date. We used a generalized linear-mixed model (GLMM) with sequential Bonferroni tests to compare the level of plant development at goose arrival time across sites. Goose identity (ID 1-19) was treated as a random-effect factor to account for the repeated measures and the site (1–5, from the southern stopover site to the breeding site) was modelled as a fixed effect.

## Author Contributions

Y.S., W.F.D., P.G., R.C.Y. and H.H.T.P. designed the research. Y.S. and Q.X. analysed the data. Y.S. wrote the first draft, and the manuscript was reviewed by all the authors.

## Supplementary Material

Supplementary Information Do Arctic breeding geese track or overtake a green wave during spring migration?
