# Supplementary material for: Do Arctic breeding geese track or overtake a green wave during spring migration?
Source: Sci Rep. 2015 Mar 4;5:8749. doi: 10.1038/srep08749 (PMC4348666; doi:10.1038/srep08749)
Supplement: Supplementary Information — Do Arctic breeding geese track or overtake a green wave during spring migration? [file srep08749-s1.pdf]

Do Arctic breeding geese track or overtake a green wave during spring migration?

Yali Si[1,2], Qinchuan Xin[1], Willem F. de Boer[3], Peng Gong[1*], Ronald C. Ydenberg[4], Herbert H. T. Prins[3]

Supplementary Information: Table S1 and S2

Table S1 Spring stopover schedule (the arrival date) of 19 female Barnacle geese derived from Eichhorn et al. (2006)[38]. Sites are: 1 - Gotland, 2- Estonia, 3 - the mouth of the river Divna, 4 - the Kanin Peninsula, and the breeding site 5 - Kolokolkova Bay. The 7 dates underlined at site 5 were filled by the mean date (June 10) of the first sighting of another 80 ringed geese and the remaining 12 dates were obtained from direct observations. The arrival time at site 3 was calculated by adding 1 day of flight after departing from the Baltic. The arrival time at site 4 was derived as the midpoint between the calculated arrival at site 3 and the (inferred and observed) arrival at site 5. * Extreme date excluded from further analyses.

GooseID\Site	1	2	3	4	5
1		3/24*	5/15	5/27	6/7
2	5/8	5/16	5/19	5/30	<u>6/10</u>
3	5/5	5/21	5/25	6/4	6/14
4	5/3	5/8	5/22	6/1	6/11
5	5/9	5/12	5/15	5/24	6/2
6	5/9	5/11	5/25	5/26	5/27
7	5/10	5/18	5/20	5/30	6/9
8	5/13	5/15	5/18	5/30	6/11
9	5/14	5/16	5/18	5/29	<u>6/10</u>
10		5/14	5/18	5/31	6/14
11		5/14	5/18	5/29	<u>6/10</u>
12	5/14	5/16	5/18	5/29	<u>6/10</u>
13	5/14	5/15	5/18	5/28	6/7
14	5/14	5/16	5/17	5/29	<u>6/10</u>
15		5/14	5/18	5/29	<u>6/10</u>
16		5/15	5/23	5/30	6/7
17	5/15	5/16	5/17	5/29	<u>6/10</u>
18	5/17	5/18	5/23	6/1	6/11
19	5/17		5/19	5/30	6/11

Table S2 Pairwise comparisons between the plant development levels at goose arrival time across different sites evaluated using the generalized linear mixed model (GLMM) with sequential Bonferroni tests. Site 1-4 indicates 4 stopover sites from south to north Gotland, Estonia, the mouth of the river Divna, and the Kanin Peninsula. Site 5 indicates the breeding site Kolokolkova Bay.

Site	Contrast Estimate	Std. Error	t	d.f.	Adj. Sig.	95% C.I.	
						Lower	Upper
1-2	15.073	1.448	10.412	83	<0.001	10.897	19.248
1-3	30.201	1.412	21.386	83	<0.001	26.18	34.222
1-4	42.878	1.412	30.364	83	<0.001	38.916	46.814
1-5	42.595	1.412	30.164	83	<0.001	38.7	46.419
2-3	15.128	1.331	11.369	83	<0.001	11.531	18.721
2-4	27.806	1.331	20.896	83	<0.001	24.298	31.314
2-5	27.523	1.331	20.684	83	<0.001	24.125	30.92
3-4	12.678	1.288	9.84	83	<0.001	9.529	15.826
3-5	12.395	1.288	9.62	83	<0.001	9.454	15.336
4-5	-0.283	1.288	-0.22	83	0.827	-2.846	2.28

Supplementary Information: Figure S1, S2, and S3

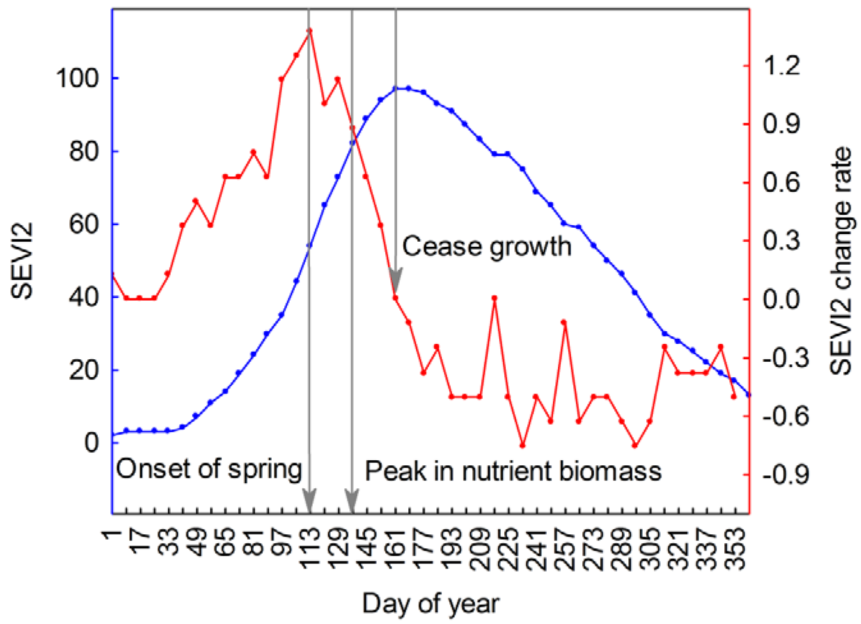

Figure S1 Calculating the date of the onset of spring and the peak in nutrient biomass. Dates were identified based on the change rate of the smoothed standardized two-band Enhanced Vegetation Index (SEVI2).

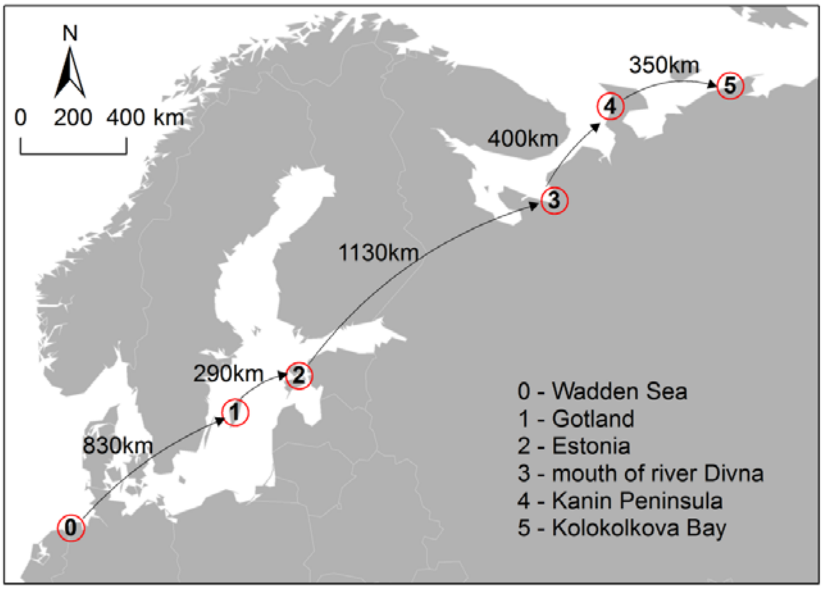

Figure S2 Spring stopover patterns and flight distances of 19 female Barnacle Geese. Locality information was derived from Eichhorn et al. (2006)[38] and the map was produced using ArcGIS 10.0 (www.esri.com). Geese left the wintering site at the Wadden Sea, stopped over at Gotland, Estonia, the mouth of the river Divna, and the Kanin Peninsula (individual geese not necessarily used each stopover site), and eventually reached the breeding site Kolokolkova Bay.

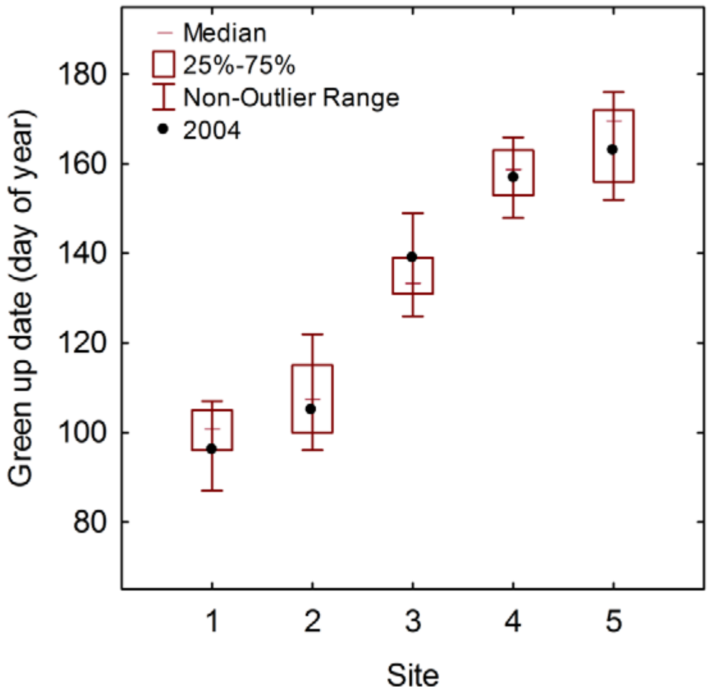

Figure S3: Statistical summary of the 10-year green-up dates (2001-2010). Dates are derived from the yearly 500 m MODIS Land Cover Dynamics product (MCD12Q2) at 4 stopover sites (1-4) and one breeding site (5). For each year, green-up dates are determined as the median values of a 50 × 50 km window centered on each site excluding non-herbivorous pixels. Black dots indicate the green-up dates in 2004.
